# Supplementary material for: Sustainable, Alginate-Based Sensor for Detection of Escherichia coli in Human Breast Milk
Source: Sensors (Basel). 2020 Feb 19;20(4):1145. doi: 10.3390/s20041145 (PMC7071128; doi:10.3390/s20041145)
Supplement: Supplementary file 1 [file sensors-20-01145-s001.zip › Supplementary Materials/SupplementaryMaterial1.docx]

Shown in Figure S1 is the assay development results for the Taguchi design outlined in Table 2.


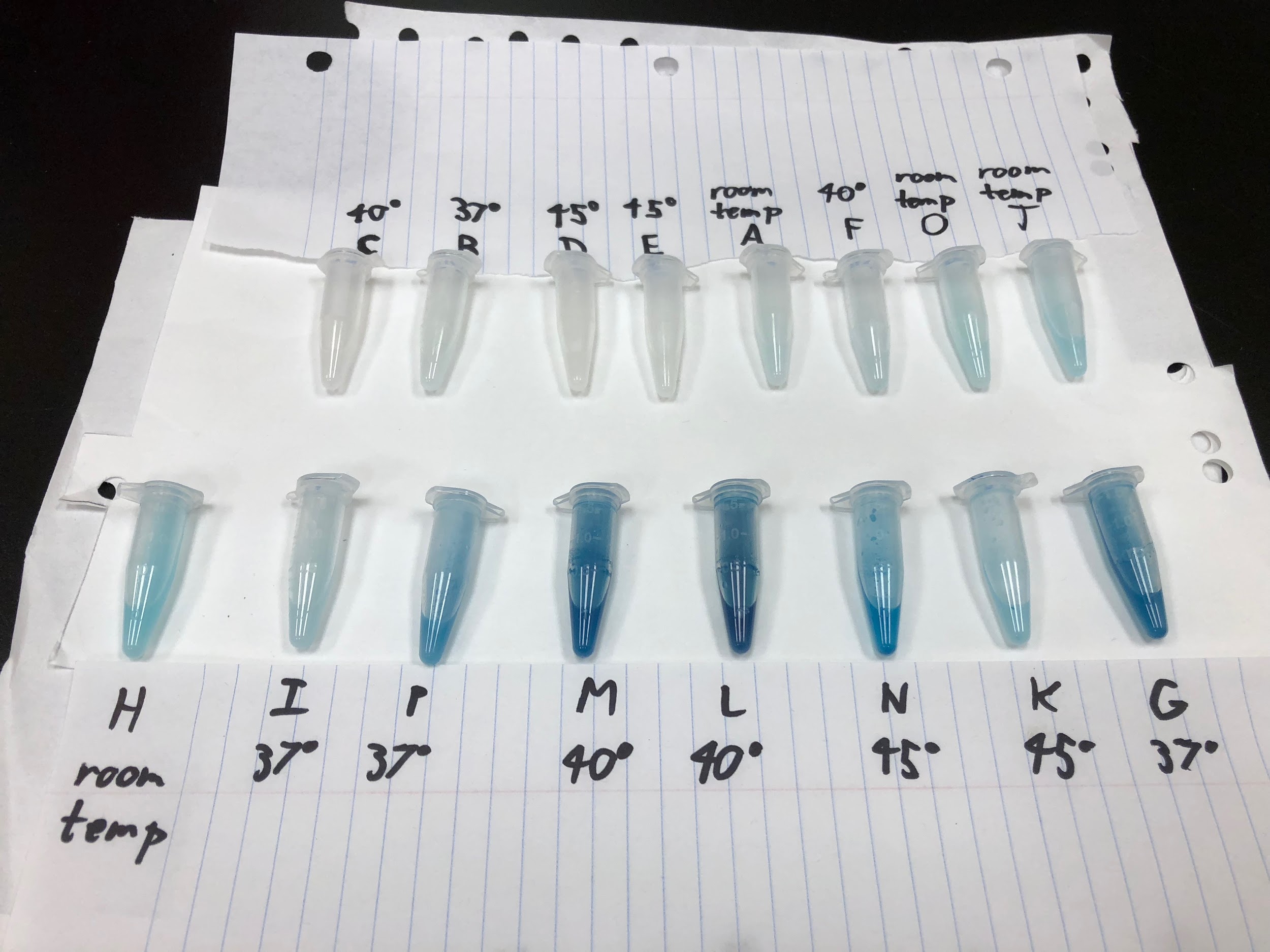


Figure S1: Samples corresponding to the Taguchi design runs analyzed using the categorical color spectrum. The labeling corresponds to the runs in Table 2. In each test tube a microcapsule was incubated with breast milk and the lysing solution. Subsequent confirmation runs were conducted using the conditions in run M.
