# Supplementary material for: Sustainable, Alginate-Based Sensor for Detection of Escherichia coli in Human Breast Milk
Source: Sensors (Basel). 2020 Feb 19;20(4):1145. doi: 10.3390/s20041145 (PMC7071128; doi:10.3390/s20041145)
Supplement: Supplementary file 1 [file sensors-20-01145-s001.zip › Supplementary Materials/SupplementaryMaterial2.docx]

Shown in Figure S2 are the data and corresponding images for the LOD determination as well as the time course of the reaction in the assay development.


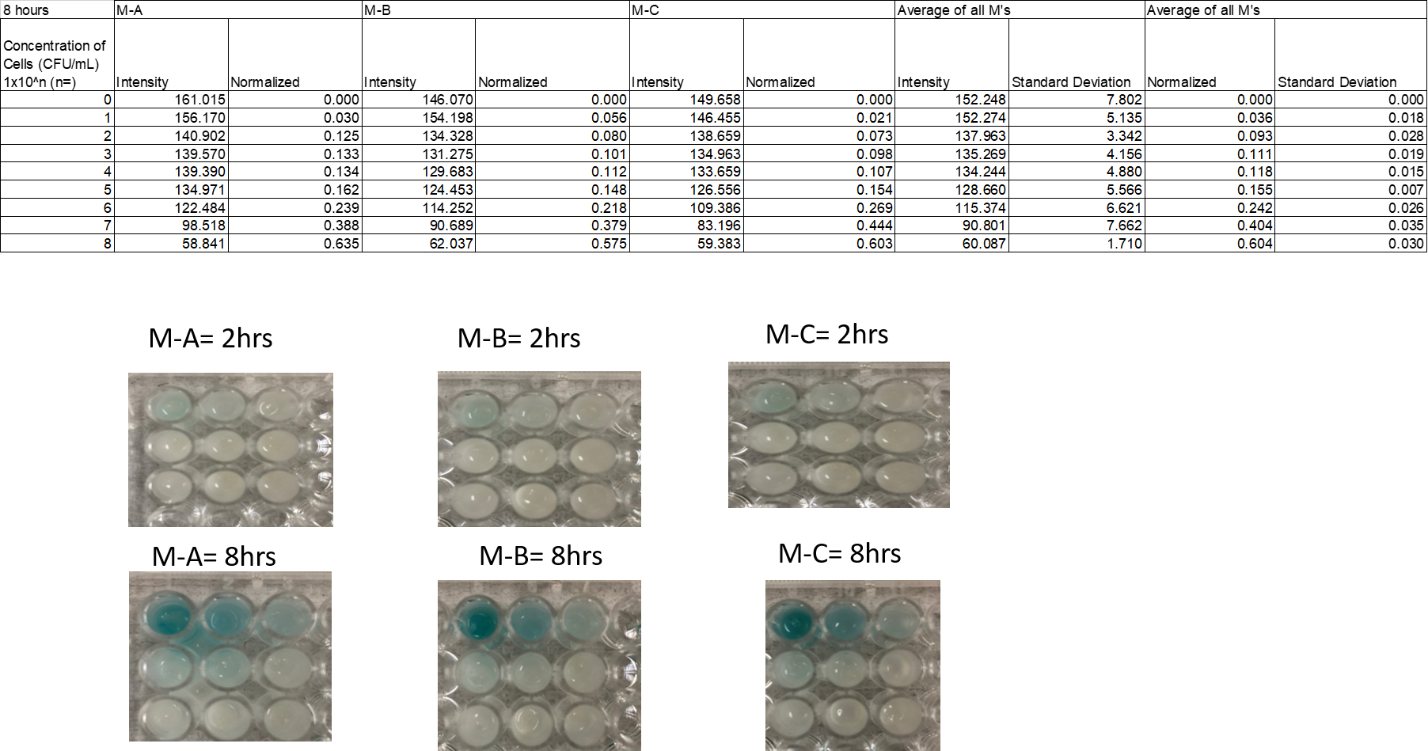


Figure S2: Raw data for the LOD determination after 8 hours of assay development and corresponding images prior to feature extraction in Image J and intensity normalization (bottom row). A single batch of bacteria, using a single batch of breastmilk was used to triplicate (M-A, M-B, M-C) the results under the conditions of run M using a concentration ladder ranging from [0-10^8^ ] CFU/mL . The images for the 2 hours of assay development are also shown (top row) but have not been used for LOD determination.
